# Authors should know the ethical rules of publication

**DOI:** 10.4103/0971-4065.62087

**Published:** 2010

**Authors:** Fahmi Yousef Khan

**Affiliations:** Department of Medicine, Hamad General Hospital, Doha, Qatar

Sir,

I read with interest an article entitled “Severe Rhabdomyolysis and Acute Renal Failure Secondary to use of Simvastatin in Undiagnosed Hypothyroidism” in your journal, the “Indian Journal of Nephrology,[1] but I was shocked when I read the same case report “Severe Rhabdomyolysis and Acute Renal Failure Secondary to use of Simvastatin in Undiagnosed Hypothyroidism,” by the same author in the “Saudi Journal of Kidney Diseases and Transplantation.”[2] Earlier, this author had published another article entitled “Profile of Stroke in a Teaching University Hospital in the Western Region,” in two different journals.[3,4]

In fact, “Shotgunning”- the simultaneous submission of essentially the same article to more than one journal is unethical because it may lead to duplicate publication. Even when one journal accepts the shot-gunned article and its authors withdraw their submission to the other journal, this practice is at least inconsiderate because it is imposed on reviewers and creates unnecessary work for the editorial staff of the journal or journals from which the paper is ultimately withdrawn.[5] Further, “Duplicate publication”- The publication of the same article in more than one journal is also unethical; it reflects the researcher's ethics and an audit of the local or regional Ethics Committee that approved it.

In conclusion, as authors, we need to be aware of the ethical rules of publication by familiarizing ourselves with all policies regarding publication at our home institutions.
